# Iron Metabolism in Oligodendrocytes and Astrocytes, Implications for Myelination and Remyelination

**DOI:** 10.1177/1759091420962681

**Published:** 2020-09-30

**Authors:** Veronica T. Cheli, J. Correale, Pablo M. Paez, Juana M. Pasquini

**Affiliations:** 1 Department of Pharmacology and Toxicology, Jacobs School of Medicine and Biomedical Sciences, Hunter James Kelly Research Institute, The State University of New York, University at Buffalo, Buffalo, New York, United States; 2 Fleni, Buenos Aires, Argentina; 3 CONICET, Instituto de Química y Fisicoquímica Biológicas, Universidad de Buenos Aires, Buenos Aires, Argentina

**Keywords:** oligodendrocytes, astrocytes, iron, transferrin, transferrin receptor, DMT1, ferritin, myelination, remyelination

## Abstract

Iron is a key nutrient for normal central nervous system (CNS) development and function; thus, iron deficiency as well as iron excess may result in harmful effects in the CNS. Oligodendrocytes and astrocytes are crucial players in brain iron equilibrium. However, the mechanisms of iron uptake, storage, and efflux in oligodendrocytes and astrocytes during CNS development or under pathological situations such as demyelination are not completely understood. In the CNS, iron is directly required for myelin production as a cofactor for enzymes involved in ATP, cholesterol and lipid synthesis, and oligodendrocytes are the cells with the highest iron levels in the brain which is linked to their elevated metabolic needs associated with the process of myelination. Unlike oligodendrocytes, astrocytes do not have a high metabolic requirement for iron. However, these cells are in close contact with blood vessel and have a strong iron transport capacity. In several pathological situations, changes in iron homoeostasis result in altered cellular iron distribution and accumulation and oxidative stress. In inflammatory demyelinating diseases such as multiple sclerosis, reactive astrocytes accumulate iron and upregulate iron efflux and influx molecules, which suggest that they are outfitted to take up and safely recycle iron. In this review, we will discuss the participation of oligodendrocytes and astrocytes in CNS iron homeostasis. Understanding the molecular mechanisms of iron uptake, storage, and efflux in oligodendrocytes and astrocytes is necessary for planning effective strategies for iron management during CNS development as well as for the treatment of demyelinating diseases.

## Introduction

Iron is an essential mineral for proper neurogenesis and myelination. Iron deficiency is the most common nutrient deficiency in humans. According to the World Health Organization, it affects more than 2 billion people and nearly half of the pregnant women worldwide (Radlowski and Johnson, 2013). Iron deficiency in late-prenatal and early-postnatal periods can lead to long-term neurobehavioral deficits in humans, including impairments in learning and memory (Lozoff et al., 2006). In contrast, excessive brain iron levels have been associated with neurodegenerative diseases (Zecca et al., 2004; Molina-Holgado et al., 2007). Iron dyshomeostasis has become a molecular signature associated with aging which is accompanied by progressive decline in cognitive processes. High iron levels in the brain coincide with neuroinflammation, neurodegeneration, and neurobehavioral deficits in almost all neurodegenerative diseases. Thus, understanding how the different cells types of the central nervous system (CNS) incorporate and manage iron through maturity or under pathological situations is critical to develop new approaches to amend cognitive dysfunctions in individuals who experienced brain iron unbalance.

Iron is an essential trophic factor that is required for oxygen consumption and ATP production (Connor and Menzies, 1995). Thus, it is an indispensable nutrient for normal brain development. Because of their elevated metabolic needs associated with the process of myelination, oligodendrocytes are the cells with the highest iron levels in the brain (Connor and Menzies, 1996). Iron accumulation by oligodendrocyte progenitor cells (OPCs) is an early event in the development of these cells; however, how OPCs and mature myelinating oligodendrocytes incorporate and manage iron is not completely understood. Furthermore, the primary source of iron for OPCs and mature oligodendrocytes during early postnatal development or in pathological situations, such as demyelination and remyelination, is currently unknown.

Astrocytes are the most abundant glial cells in the brain and are strategically located to acquire nutrients from the circulating blood such as iron (Abbott et al., 2006). Astrocytic end-feet processes form intimate contacts with the abluminal side of brain capillary endothelial cells (BCECs) in all brain regions, and this close relationship is generally believed to denote an important role for astrocytes in maintaining the blood–brain barrier (BBB) integrity (Abbott et al., 2006; Price et al., 2018). Thus, the astrocytic end-feet processes could play an important metabolic role by transporting nutrients from the BCECs into the brain. However, the mechanisms of iron uptake and efflux in astrocytes during postnatal brain development and under pathological situation such as brain demyelination are relevant questions in the field and are currently under investigation.

Here, we will review our current understanding of how oligodendrocytes and astrocytes participate in brain iron balance and distribution. We divide the review into two principal components: In the first, we define the key molecular regulators of iron metabolism in OPCs and mature oligodendrocytes, where data are available, how such proteins affect OPC development and oligodendrocyte function. In the second part, we discuss how iron metabolism in astrocytes in turn affects OPC and oligodendrocyte biology in the healthy nervous system and how dysregulation of iron homeostasis in astrocytes can disrupt tissue health and function. We finish by briefly outlining the potential use of the transferrin receptor as a means to safely deliver drugs to the CNS.

## Iron Homeostasis in Oligodendrocyte Development

Oligodendroglial cells require iron as a cofactor for several enzymes involved in the proliferation and differentiation of OPCs, and in the production of cholesterol and phospholipids, which are essential myelin components (Stephenson et al., 2014). OPCs as well as mature myelinating oligodendrocytes are the highest iron-rich cells in the brain (Connor and Menzies, 1996). Iron concentration in neurons and glial cells was recently determined in adult rat brains using proton induced X-ray spectrometry (Reinert et al., 2019). It was found that 0.57 mM was the average intracellular iron concentration in cortical neurons, and that astrocytes and microglia cells presented greater iron levels, with averages of 1.29 mM and 1.76 mM, respectively (Reinert et al., 2019). However, the iron concentration found in mature oligodendrocytes was 3.05 mM, which double the iron content of astrocytes and microglia cells and quintuples the average in neurons (Reinert et al., 2019). Physiological iron accumulation occurs in the CNS mainly in oligodendroglial cells and thus OPCs stained for iron more strongly than any other cell in the postnatal mouse brain (Benkovic and Connor, 1993; Connor and Menzies, 1995; Connor et al., 1995). In rodents, myelination starts in the first postnatal week, peaks around postnatal day 30 (P30) and is almost complete by P60 (Benjamins and Morell, 1978). The speed of myelin production correlates very well with changes in iron concentrations in several brain regions (Roskams and Connor, 1994; Tarohda et al., 2004). For example, rising iron concentrations were found in the mouse brain during early postnatal development, reaching adult levels by P70 (Tarohda et al., 2004). These data suggest that during the first 2 postnatal months, OPCs and mature oligodendrocytes need to develop all the necessary iron related mechanisms and regulatory circuits to safely incorporate and accumulate elevated amounts of iron.

**Figure 1.**
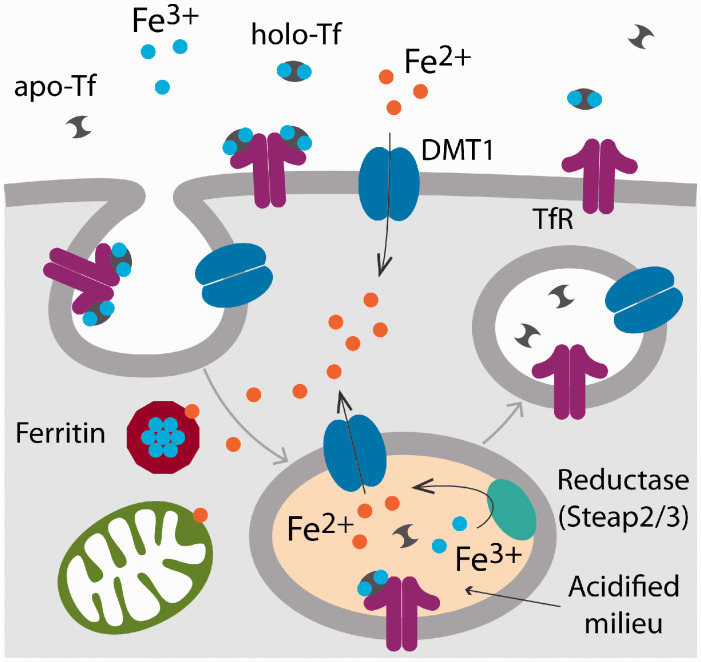
Schematic Representation of the Tf Cycle. Fe^3+^ in the brain interstitial fluid binds to apo-Tf to form holo‑Tf. Holo‑Tf binds to TfR on the cell surface. The TfR–holo-Tf complex undergoes endocytosis through clathrin pit formation. The endosome then acidifies, and the endosomal metalloreductase reduces Fe^3+^ to Fe^2+^, allowing iron, now released from Tf, to be transported into the cytosol by DMT1. Iron can then bind to chaperones that donate iron to specific proteins (not shown), enter mitochondria, or be stored in ferritin. At the plasma membrane, DMT1 can uptake Fe^2+^ independent of the Tf cycle. Finally, apo-Tf and the TfR are recycled back to the luminal membrane. DMT1: divalent metal transporter 1.

Several proteins work together to maintain cell iron homeostasis by regulating iron uptake and storage. Transferrin (Tf, *TF*) is the most important iron carrier molecule, is mainly synthesized in the liver, and has the capacity to bind two atoms of ferric iron with high efficiency. Tf loaded with iron (holo-Tf), binds with great affinity to the Tf receptor (TfR, *TFRC*) on the cell surface, after which the Tf-TfR complex is internalized through receptor-mediated endocytosis (Figure 1). Under the acid interior milieu of the endosome, ferric iron is next reduced to ferrous iron by metalloreductases such as Steap2 and 3 (*STEAP2, STEAP3*) and is transported across the endosomal membrane to the cytosol by the divalent metal transporter 1 (DMT1, *SLC11A2*; Garrick et al., 2003; Figure 1). In the cytosol, iron may be transported by binding to chaperones that donate iron to specific target proteins, or it may traffic to the mitochondria. Inside the endosome, the affinity of Tf without iron (apo-Tf) to its receptor significantly drops, resulting in the dissociation of the Tf-TfR complex (Figure 1). In the final step of this cycle, apo-Tf and the TfR are recycled back to the luminal membrane (Figure 1). Tf is synthesized mainly in the liver and is secreted into the blood stream from where it is able to cross the BBB (Broadwell et al., 1996). However, the amount that effectively reaches the brain parenchyma is considered to be insufficient to satisfy the CNS metabolic demands. It has been shown that premyelinating oligodendrocytes and epithelial cells of the choroid plexus synthesize and release apo-Tf inside the CNS (Bloch et al., 1985; Espinosa de los Monteros et al., 1994). In fact, the brain is the only organ in which the expression of Tf increases after birth (Escobar-Cabrera et al., 1994, 1997). Apo-Tf produced by oligodendrocytes and epithelial cells of the choroid plexus probably binds ferric iron in the brain parenchyma. This ferric iron could be released by astrocytes and microglial cells (Z. Chen et al., 2019); thus, this could be a central mechanism by which iron is distributed in the CNS throughout development.

It is very well known that iron deficiency during perinatal brain development significantly affects oligodendrocyte maturation, causing long-lasting hypomyelination which continues into adulthood, even after restoration of normal iron levels in the diet. Elevated numbers of OPCs were found in several brain areas under iron deprivation conditions (Badaracco et al., 2008). These OPCs were capable to proliferate and migrate but unable to mature and generate normal quantities of myelinating oligodendrocytes. Importantly, hypomyelination induced by perinatal iron deprivation was found to be ameliorated by apo-Tf treatment (Badaracco et al., 2008). At several postnatal time points, the production of myelin proteins and the number of mature myelinating oligodendrocytes in iron-deficient mice was found to be almost normalized by a single intracranial injection of apo-Tf (Badaracco et al., 2008). In the same line, several groups have shown that increased levels of Tf in the brain of perinatal rats promote the synthesis of myelin proteins (Espinosa de los Monteros et al., 1988; Escobar-Cabrera et al., 1994, 1997), and that Tf overexpression in the CNS of mice significantly increases the production of myelin components such as galactolipids and phospholipids, which form structurally normal myelin sheets (Saleh et al., 2003).

Tf also promotes the development of OPC *in vitro* (Raff et al., 1983; Paez et al., 2002), and the maturation of the oligodendroglial cell lines N19 and N20 (Paez et al., 2004, 2005, 2006). The overexpression of Tf in these two cells lines induces a dramatic morphological differentiation and stimulates the interaction of these cells with cortical neurons in a coculture system (Paez et al., 2005, 2006). High concentration of apo-Tf in the culture media of OPCs isolated from iron-deficient pups also promotes the maturation of these cells to control levels (Badaracco et al., 2008). Interestingly, apo-Tf enhances the effect of the thyroid hormone on OPC maturation and in turn, the thyroid hormone promotes the synthesis of apo-Tf in OPCs (Marziali et al., 2015, 2016). The effects of apo-Tf on oligodendrocyte maturation and myelination were found to be mediated by several signaling pathways. Tf activates the cyclic adenosine monophosphate pathway and the expression of several genes related to mitochondrial function and lipid metabolism in cultured OPCs (Marta et al., 2002; Garcia et al., 2007). Studies in primary oligodendrocytes have also demonstrated that the Fyn/MEK/ERK and PI3K/Akt pathways are strongly activated after apo-Tf treatment (Perez et al., 2009, 2013). Importantly, OPC incorporate Tf through clathrin-heterotetrameric adaptor protein 2-mediated endocytosis, which indicates that, during iron delivery, Tf binds to its cognate receptors on the cell surface and enters the endosome/lysosome canonical pathway through clathrin-coated vesicles (Perez et al., 2013). While the precise mechanisms underlying OPC maturation in response to apo-Tf require further investigations, as well as the participation of iron and the TfR in this process, these data suggest that several intracellular pathways are involved.

Like holo-Tf, apo-Tf is also able to bind to the TfR at serum pH, forming short-lived complexes (Kleven et al., 2018); this suggests that at low systemic iron levels apo-Tf can efficiently compete with holo-Tf for binding the TfR. Several noncanonical functions of the TfR have been defined. TfR interacts with Fth and modulates the expression of hepcidin (*HAMP*), an iron-regulatory hormone in hepatocytes (Schmidt et al., 2008). It has been shown that phosphorylation of TfR by Src kinases promotes cell survival (Jian et al., 2011) and that TfR activates the JNK and ERK signaling pathways through interactions with stearic acids (Kasibhatla et al., 2005; Senyilmaz et al., 2015). Furthermore, TfR was implicated in the homeostatic maintenance of the intestinal epithelium, acting through a function that is independent of its role on iron incorporation (A. C. Chen et al., 2015). Thus, the interaction of apo- and holo-Tf with TfR might result in the activation of intracellular signaling cascades in oligodendrocytes independent of the role of these proteins in iron uptake. The participation of these signaling pathways in OPC and oligodendrocyte development, and whether these intracellular cascades are modulated by the TfR in oligodendrocytes, needs to be defined in future studies.

Although the Tf cycle is assumed to be the general mechanism for OPC iron uptake, this has not been validated experimentally. In the CNS, previous studies have shown that the TfR is mainly expressed in endothelial cells of the brain capillaries and neurons but not in OPCs and oligodendrocytes (Roberts et al., 1993). However, some works indicate that the TfR is present in OPCs and then is down regulated in mature myelinating oligodendrocytes. For example, TfR expression in OPCs was found to be undetectable when cells were allowed to differentiate into mature oligodendrocytes *in vitro* (Li et al., 2013). In agreement, an RNA-sequencing transcriptome analysis of glial cells performed by Y. Zhang et al. (2014) has revealed that the TfR is present in OPCs, peaks in newly formed oligodendrocytes and then, it is downregulated in mature myelinating cells. These data suggest the existence of a developmental time window in which oligodendroglial cells uptake iron via the Tf cycle. DMT1 participates in the Tf cycle by transporting iron out of the endosomes (Garrick et al., 2003); thus, DMT1 is essential to the oligodendrocyte Tf cycle (Figure 1). Cheli et al. (2018) have recently established that DMT1 is crucial for proper oligodendrocyte maturation and is required for an efficient remyelination of the adult brain. Blocking DMT1 synthesis in primary cultures of OPCs reduced iron uptake and significantly delayed OPC development (Cheli et al., 2018). In addition, a significant hypomyelination was found in DMT1 conditional knock-out mice in which DMT1 was postnatally deleted in NG2 or Sox10 positive OPCs. The brain of DMT1 knock-out animals showed a decrease in the expression levels of myelin proteins and a substantial reduction in the percentage of myelinated axons. This reduced postnatal myelination was accompanied by a decrease in the number of myelinating oligodendrocytes and with a rise in proliferating OPCs (Cheli et al., 2018). These results indicate that DMT1 is crucial for OPC maturation and for the normal myelination of the mouse brain. However, most cells are capable also of non-Tf-bound iron uptake through DMT1 (Garrick et al., 2003). This could be an alternative route of iron assimilation in OPCs and mature oligodendrocytes independent of the Tf cycle (Figure 1). Determining which one of these two mechanisms is the most active in OPCs/myelinating oligodendrocytes and how these two possible alternatives of iron absorption change during the development of the CNS should be the subject of future investigations.

Ferritin is the primary intracellular iron-storage protein in most cells and is essential for keeping iron in a soluble and nontoxic form. Ferritin is a 24-subunit heteropolymer, composed of Heavy (Fth, *FTH1*) and Light (Ftl, *FTL*) chains, capable of binding over 4,500 atoms of iron (Harrison and Arosio, 1996), which can be mobilized for metabolic purposes through lysosomal turnover (Y. Zhang et al., 2010). The Fth subunit contains ferroxidase activity, which is required for converting soluble ferrous into ferric iron which is then deposited inside the ferritin core (Harrison and Arosio, 1996). As a result of its ability to sequester iron rapidly, Fth is considered an antioxidant protein (Balla et al., 1992) because iron removed quickly by this protein is not available for inducing oxidative damage. For example, increased cytoplasmic iron levels and reactive oxygen species (ROS) formation were found in Fth knock-out fibroblasts, which significantly reduce the viability of these cells *in vitro* (Darshan et al., 2009). Fth appears to have a similar protective role in oligodendroglial cells. For instance, Fth synthesis increased in immature oligodendrocytes following exposure to hypoxic conditions or tumor necrosis factor alpha (TNF-α; Qi and Dawson, 1994; Sanyal and Szuchet, 1995). Thus, the vulnerability of oligodendroglial cells to oxidative damage and the expression of Fth are connected. In the absence of Fth, OPCs and mature oligodendrocytes may experience an increase in free cytoplasmic iron, ROS formation, and oxidative stress. Interentingly, Fth deletion in mature myelinating oligodendrocytes causes neuronal loss and oxidative damage in mice (Mukherjee et al., 2020). Mukherjee et al. (2020) have established that Fth secreted by mature oligodendrocytes is required for the protection against iron-mediated axonal damage in the adult brain. Although the mechanism of Fth secretion in oligodendrocytes is not completelly understood, these data point to a role of myelinating oligodendrocytes in providing an antioxidant defense system to support neurons against iron-mediated cytotoxicity.

**Figure 2.**
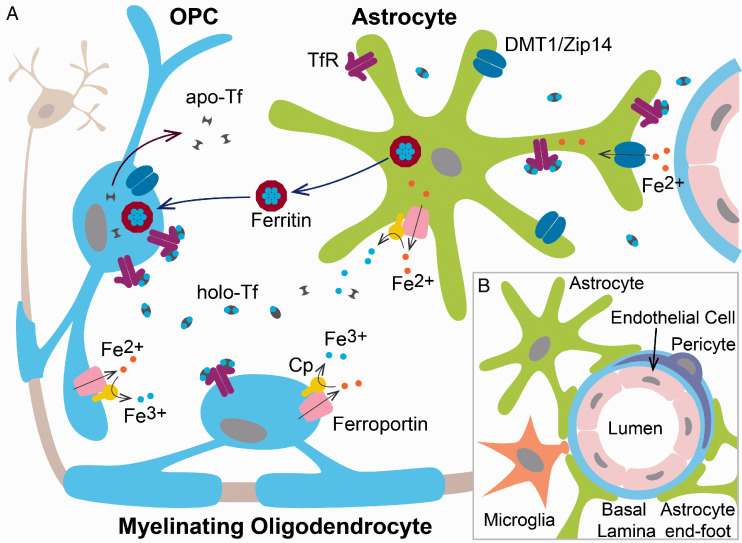
A: Schematic illustration of glial iron metabolism. This hypothetical model illustrates the possible interactions between oligodendrocytes and astrocytes in iron metabolism during development as well as in the adult brain. (1) Most Tf in the brain is synthesized and secreted by OPCs and oligodendrocytes as apo‑Tf. (2) Astrocytes, OPCs and mature oligodendrocytes probably acquire most iron through TfR and holo‑Tf that is present in interstitial fluid and cerebrospinal fluid. (3) DMT1 can participate in Fe^2+^ absorption independent of the Tf cycle. (4) Ceruloplasmin can oxidize Fe^2+^ to Fe^3+^ and then promote ferroportin-mediated Fe^2+^ release in astrocytes, OPCs and mature oligodendrocytes. (5) Iron is stored in astrocytes, OPCs and myelinating oligodendrocytes mainly in the form of ferritin. Ferritin released from astrocytes could be the main mechanism of OPC and matue oligodendrocyte iron intake. B: Schematic representation of the neurovascular unit organization. DMT1: divalent metal transporter 1; OPC: oligodendrocyte progenitor cells.

Different cell types in the brain have differing ferritin compositions. Neurons express mainly Fth, whereas microglia express mostly Ftl. Oligodendroglial cells are the only cell type of the CNS that have an equal combination of both subunits (Connor, 1994; Connor and Menzies, 1995). The fact that oligodendrocytes have an equal mixture of both ferritin chains illustrates the high usage of iron by these cells. RNA sequencing of the oligodendrocyte lineage done by Y. Zhang et al. (2014) found that ferritin subunits are highly expressed in OPCs and their expression increases as these cells develop. It has been also shown that the accumulation of iron coincides with the appearance of ferritin in mature oligodendrocytes in the developing brain (Blissman et al., 1996; Sanyal et al., 1996), and that myelination is significantly affected in Fth heterozygous knock-out mice (Ortiz et al., 2004). Ferritin was found to be significantly upregulated during OPC maturation *in vitro* as well as *in vivo*, (Li et al., 2013; Y. Zhang et al., 2014), and Roskams and Connor (1994) showed that the expression of ferritin in the rodent brain is highest at birth and declines to minimum levels after the third postnatal week. This indicates that iron storage by ferritin is important for OPC maturation during the first postnatal weeks. Importantly, it has been suggested that during development OPCs mainly uptake iron via ferritin endocytosis (Todorich et al., 2008, 2011; Rouault, 2013). Oligodendroglial cells bind Fth and that Fth binds to white matter tracts *in vivo* (Todorich et al., 2008). In mice, the T cell immunoglobulin and mucin domain-containing protein-2 (Tim-2) was proposed to be the specific receptor by which oligodendrocytes internalize Fth (Todorich et al., 2008). It was shown *in vitro* that iron-loaded microglial cells release Fth into the culture media which stimulates oligodendrocyte survival (X. Zhang et al., 2006). It is tempting to speculate that during brain development microglial cells and astrocytes secrete iron-loaded Fth to distribute iron among cells of the CNS (Figure 2A). This hypothesis could be tested *in vivo* by using conditional knock-out mice for Tim-2 and Fth in oligodendroglial cells, microglial cells, and astrocytes.

## Oligodendrocyte Iron Metabolism in Myelin Injury and Repair

Iron is required for many enzymes that influence the metabolism, proliferation, and differentiation of oligodendroglial cells. Therefore, iron deficiency in OPC and mature oligodendrocytes would likely negatively affect the remyelination process (Stephenson et al., 2014), and iron availability in the demyelinated brain parenchyma might be a key factor for an efficient remyelination. However, how new generated OPC incorporate and store fresh iron through the progression of the remyelination process is not known. Iron metabolism has been shown to be disrupted in neurodegenerative and demyelinating diseases. For example, magnetic resonance imaging (MRI) and histological studies in multiple sclerosis (MS) patients have revealed that demyelination leads to accumulation of iron in gray matter and iron depletion in normal appearing white matter (Rouault, 2013). As well, abnormal CNS iron deposits were found in both, gray and white matter structures in several animal models of MS. For instance, iron staining is typically associated with vessels and reactive astrocytes and microglial cells in experimental autoimmune encephalomyelitis (EAE) mice (Forge et al., 1998; Pedchenko and LeVine, 1998). These features are observed during the active stage of the disease as well as during the recovery phase (Forge et al., 1998). Still, the mechanism by which astrocytes and microglial cells release this iron back to the brain parenchyma, and if this iron is, to some extent, available to new OPCs during remyelination is unknown.

It has been established that apo-Tf treatment significantly promotes remyelination and OPC differentiation in a rat model of demyelination induced by cuprizone (Adamo et al., 2006). A single intracranial injection of apo-Tf at the beginning of the remyelination phase of the cuprizone model of demyelination (Armstrong et al., 2002; Paez et al., 2012) was associated with a clear increase in myelin production (Adamo et al., 2006). This phenomenon was complemented with a marked expansion in the number of proliferating OPCs and mature myelinating oligodendrocytes throughout the entire recovery process (Adamo et al., 2006). In addition, intraventricular apo-Tf treatment promotes the remyelination of lysolecithin lesions in rats, through a mechanism mediated by the Notch signaling pathway (Aparicio et al., 2013). In the subventricular zone as well as in the corpus callosum, Notch activation promotes both OPC proliferation and the commitment of neural progenitor cells toward the oligodendroglial progeny (Aparicio et al., 2013). However, whether or not these effects are mediated by iron-bound to Tf, apo-Tf, or a combination of both, is an open question. It is possible that high concentrations of apo-Tf in the brain parenchyma stimulate the redistribution of iron in the demyelinated CNS, helping new generated OPCs to uptake fresh iron during remyelination. In the same line, the role of DMT1 in demyelination and remyelination was recently tested using the cuprizone model of myelin injury and repair. Cheli et al. (2018) have found that after demyelination, DMT1 is critical for newly generated OPCs to import iron and efficiently remyelinate demyelinated axons (Cheli et al., 2018). DMT1-deficient OPCs mature more slowly than control cells and were less effective in remyelinating several structures of the adult brain including the corpus callosum and cortex. After 2 weeks of recovery, conditional knock-out mice for DMT1 in OPCs presented a marginal increase in the percentage of remyelinated axons with an important reduction in the myelin thickness (Cheli et al., 2018). These findings reveal the inability of DMT1 knock-out OPCs to efficiently remyelinate demyelinated axons and suggest that iron levels and iron redistribution during demyelination can significantly influence the effectiveness of the remyelination process. It is likely that proteins such as DMT1 and Tf implicated in OPC differentiation and maturation may induce positive signals for recovery.

White matter damage after hypoxia/ischemia (H/I) occurs in the immature brain and leads to periventricular leukomalacia (PVL), a neurological condition associated with periventricular brain injury in the premature infant (Volpe, 2001). Lethal injury to OPCs in the cerebral white matter was proposed to be a key feature in PVL resulting in hypomyelination (Volpe, 2001; Haynes et al., 2003). The potential beneficial effect of apo-Tf in this condition was tested by Guardia-Clausi et al. (2010, 2012) using a neonatal rat model of white matter damage by H/I. In this model, the intranasal delivery of apo-Tf promotes the survival and maturation of OPCs and stimulates the myelination of several brain areas. Particularly, apo-Tf treatment increased the number of OPCs and their survival in the neonatal subventricular zone after a H/I insult (Guardia-Clausi et al., 2010, 2012). These observations indicate a role for apo-Tf as a potential inducer of OPCs in the neonatal mouse brain in acute demyelination caused by H/I. Furthermore, these results suggest that the intranasal administration of apo-Tf can be used as a noninvasive method for the clinical treatment of demyelinating hypoxic-ischemic events.

## The Participation of Astrocytes in Brain Iron Balance

The BBB is a diffusion barrier that impedes the influx into the brain parenchyma of certain molecules on the basis of polarity and size (Abbott et al., 2006). The principal cellular constituents of the BBB are cerebral capillary endothelial cells that form tight junctions and are surrounded by a basal lamina and perivascular pericytes (Ballabh et al., 2004). In addition, the vasculature of the brain is almost completely ensheathed by astrocytic processes called astrocytic end-feet. These astrocytic end-feet processes are specialized units that function to maintain the ionic and osmotic homeostasis of the brain (Amiry-Moghaddam et al., 2003; Simard and Nedergaard, 2004). It has been hypothesized that astrocytes are an essential component of the so-called neurovascular unit formed between BCECs and pericytes (Figure 2B; Abbott et al., 2006; Price et al., 2018), and that astrocytes theoretically can transport iron directly from BCECs to neurons and oligodendrocytes by means of intracellular transport (Price et al., 2018).

Like other metabolites, the entry of iron into the brain is tightly regulated by the BBB, and specific transport processes are required to import this essential nutrient into the CNS (Rouault, 2006). As discussed before, the most common mechanism involves the Tf cycle. The brain is the only organ that expresses the TfR on the apical and luminal side of its capillaries (Kawabata et al., 1999). Hence, TfR-mediated uptake of iron by BCECs can be followed by further transport into the brain. This is believed to be the major mechanism by which iron is transported across the BBB, while only a small proportion of iron is mobilized via the choroid’s plexuses (Crowe and Morgan, 1992). However, how iron is transported through BCECs is not completely understood. Iron can cross BCECs either by means of receptor mediated transcytosis of holo-Tf or by receptor-mediated endocytosis at the luminal side followed by detachment of iron from Tf inside the endosomes. Nevertheless, there is no evidence of Tf transportation through BCECs (Moos et al., 2006). DMT1 is strongly expressed in the astrocyte end-feet contacting BCECs directly (Burdo et al., 2001; X. S. Wang et al., 2001), which suggests that astrocytes can potentially uptake non-Tf-bound iron at the BBB. In this line, several factors which can release iron from holo-Tf are present in the brain extracellular fluid and may be at relatively high concentrations near the BCEC-astrocyte end-foot junction, including hydrogen ions, citrate, ATP, and other nucleotides (Montana et al., 2006). These small molecules, released from astrocytes and present in high concentrations at the brain interstitial fluid, can be essential intermediators in iron mobilization from BCECs to astrocytes.

Astrocytes show a great complexity of iron control (Jeong and David, 2006). Cultured astrocytes are capable of importing both Tf and non-Tf iron bound and express high levels of TfR and DMT1 *in vitro* (Lane et al., 2010; Tulpule et al., 2010). Furthermore, the presence of mRNAs for TfR and DMT1 has been found in acutely isolated cortical astrocytes from mouse brains (Y. Zhang et al., 2014). However, the Tf cycle is probably not the main process by which astrocytes obtain iron from endothelial cells (Schipper et al., 1999; Pelizzoni et al., 2013). ATP and other nucleotides are released from astrocytes and other brain cells (Montana et al., 2006) and could act as mediators of iron release from holo-Tf. Iron-regulated transporter-like DMT1 and ZIP14 (solute carrier family 39 member 14, *SLC39A14*), which are strongly expressed in astrocytic plasma membranes and end-feet processes, are perhaps the main players in astrocyte iron incorporation from BCECs (Figure 2A; Qian et al., 2000; Lane et al., 2010). Indeed, the *in vivo* expression of DMT1 was reported to be confined to astrocytic perivascular end-feet (Moos and Morgan, 2004). Ferroportin (*SLC40A1*) is the only known iron exporter, and it is a transmembrane protein that transports iron from inside to outside of the cell (Ward and Kaplan, 2012). Ferroportin cooperates with ceruloplasmin (*CP*), a copper-containing ferroxidase enzyme which catalyzes the oxidation of ferrous iron to ferric iron, the form of the metal that binds to Tf (Marchi et al., 2019). The ferroportin-ceruloplasmin system represents the main pathway for cellular iron efflux, and it is responsible for physiological regulation of cellular iron levels (Musci et al., 2014). Ferroportin and ceruloplasmin are highly expressed on astrocytic cell membranes, and both proteins likely play an important role in iron mobilization from these cells into the extracellular brain space (Figure 2A; Jeong and David, 2003; Z. Chen et al., 2019).

The basal iron content of cultured astrocytes is relatively low; approximately 10 nmol per milligram of cellular protein (Hoepken et al., 2004). This low level probably reflects the short physiological iron requirement of astrocytes. Astrocytes contain limited ferritin especially in the human striatum (an iron-rich area), where astrocytes mainly express Ftl (Connor, 1994). It is important to note that a small proportion of astrocytes stain for iron when brain sections are processed for the Perls staining, which labels iron stored in ferritin. This observation supports the notion that astrocytes may be mostly involved in iron trafficking and not iron accumulation. However, the number of iron-labeled astrocytes increases in the aging brain (Connor et al., 1990). This increase in intracellular iron appears to be stored in astrocytic mitochondria and Ftl (Schipper et al., 1998). Cultured astrocytes contain Fth (Papadopoulos et al., 1997; Regan et al., 2002) that is posttranscriptionally regulated by the availability of extracellular iron. Astrocytes contain low amounts of Fth, and iron depletion produced by the treatment of iron chelators results in Fth downregulation (Hoepken et al., 2004). Conversely, application of ferric ammonium citrate, hemoglobin or ferrous sulfate strongly increases Fth expression in cultured astrocytes (Regan et al., 2002; Hoepken et al., 2004). In addition to iron availability, the amount of Fth and Ftl in cultured astrocytes is upregulated by hypoxia and by reoxygenation after ischemia (Irace et al., 2005).

 Astrocytes from iron-deficient animals do not mature normally, display decreased expression of CX43 and glial fibrillary acidic protein (GFAP), and proliferate more than control astrocytes *in vitro* as well as *in vivo* (Rosato-Siri et al., 2010, 2018). It is well-known that astrocytes influence OPC maturation through the secretion of different growth factors (Jeong and David, 2006). Thus, changes in the maturational stage of astrocytes due to iron unbalance can significantly affect OPC maturation and the postnatal myelination of the CNS. On the other hand, iron efflux from astrocytes mediated by the iron exporter ferroportin was shown to be relevant for OPC maturation and myelination/remyelination (Schulz et al., 2012), which suggests that astrocytic iron uptake and release are connected with OPCs and oligodendrocytes iron homeostasis. In addition, astrocytes might also deliver iron to microglia, which require this nutrient for the synthesis of several cytokines such as interleukin-1β and TNF-α. These cytokines could then influence OPCs development directly, or indirectly, by stimulating the production and release of growth factors by astrocytes. Thus, our knowledge about the crosstalk between OPC and astrocyte iron metabolism is incomplete. More research is needed to define the molecular mechanisms of iron uptake and efflux in astrocytes during postnatal brain development as well as in the adult CNS. This could be achieved *in vivo* by using new genetic tools such as the *Cre-Lox* and the CRISPR-Cas9 systems. Describing how changes in astrocytic iron homeostasis influence OPC development and myelination will certainty shed new light on the role of glial cells in brain iron equilibrium.

## Astrocytic Iron Metabolism in Demyelinating Diseases

Growing evidence indicates that astrocytes might play a critical role in both, oligodendrocyte injury and axonal degeneration in MS, particularly during the progressive phase of the disease (A. Williams et al., 2007; Mayo et al., 2012; Correale and Farez, 2015). Indeed, astrocytes were found to be highly abnormal near MS lesions (Brosnan and Raine, 2013; Clemente et al., 2013). Astrocytes could intensify MS progression through the secretion of cytotoxic factors, which block remyelination and axonal regeneration, or by modulating the permeability of the BBB and contributing to axonal mitochondrial dysfunction (Correale and Farez, 2015). By contrast, astrocytes also stimulate myelin repair by secreting trophic factors (Arnett et al., 2001; Mason et al., 2001; R. Fischer et al., 2014) or by providing energy and substrates, such as lactate and iron, to oligodendrocytes and axons (Moore et al., 2011). Iron metabolism has been shown to be disrupted in demyelinating diseases. For example, MRI studies have shown iron accumulation in the CNS of MS patients (Bakshi et al., 2002; Neema et al., 2009). This iron accumulation occurs predominantly in infiltrating macrophages/reactive microglia and activated astrocytes (Hametner et al., 2013; Rouault, 2013). Likewise, abnormal CNS iron deposits were found in both, gray and white matter structures in several animal models of MS. For example, iron staining is typically associated with vessels and reactive microglia and astrocytes in EAE mice (Forge et al., 1998; Pedchenko and LeVine, 1998). These features are observed during the active stage of the disease as well as during the recovery phase (Forge et al., 1998) and increase with disease severity and progression (Zarruk et al., 2015).

Activated microglia and astrocytes may acquire high levels of iron by phagocytosing myelin and oligodendrocyte debris (Zamboni, 2006; Singh and Zamboni, 2009; R. Williams et al., 2011). In chronic MS lesions, activated microglia cells contain high amounts of iron. Iron accumulation in microglia cells is partially regulated by pro-inflammatory cytokines and high intracellular iron induces a persistent pro-inflammatory state in these cells (for a review see Gillen et al., 2018). Although, the role of microglial cells in demyelinating diseases is beyond the scope of this review, published data suggest that iron-positive microglia may be a significant contributor to tissue injury and disease severity in MS. Astrocytes located near EAE lesions upregulate iron importers such as TfR, DMT1, and ZIP14, which suggests that these cells possess at least three different mechanisms to actively uptake iron during demyelination (Zarruk et al., 2015). In the other hand, the multicopper ferroxidase ceruloplasmin, which promote cell iron efflux through ferroportin, is highly expressed in astrocyte membranes (Vashchenko and MacGillivray, 2013; Z. Chen et al., 2019). Iron efflux deficiency, iron overload, and increased free radical production were found in multicopper ferroxidases knock-out astrocytes (Z. Chen et al., 2019). This indicates that iron oxidases, such as ceruloplasmin, are important for the prevention of astrocyte iron accumulation and thus may be protective against oxidative damage in demyelinating disorders. The robust expression of iron influx and efflux molecules suggests that astrocytes are well equipped to take up and safely recycle iron in the CNS during demyelination (Zarruk et al., 2015). Interestingly, OPC differentiation and remyelination were impaired in a mouse model of focal chemical demyelination when ferroportin was specifically deleted in astrocytes (Schulz et al., 2012). These data highlight the importance of iron availability in the remyelinating mouse brain and suggest that iron distribution from astrocytes can significantly influence the effectiveness of the remyelination process.

Iron is essential for myelin synthesis but also has the ability to generate toxic free radicals if not properly handled. Oxidative stress is a major driving force for myelin injury. It is induced by the production of reactive oxygen and nitric oxide species, predominantly by microglia and astrocytes in response to their activation (M. T. Fischer et al., 2012). Oxidative injury is amplified by the liberation of ferrous iron from degenerating cells into the extracellular space, which can lead to the formation of highly reactive hydroxyl radicals through the Fenton reaction (Papanikolaou and Pantopoulos, 2005). Amplification of oxidative injury by iron is particularly important in inflammatory demyelinating diseases due to widespread destruction of myelin and chronic astrocyte and microglial activation. For this reason, oxidative stress is under physiological conditions limited by several antioxidant mechanisms, which are mainly detected in astrocytes and microglial cells (Lassman and Horssen, 2016). The malfunction of these antioxidant mechanisms due to high intracellular iron concentrations might induce mitochondrial injury and energy deficiency in astrocytes and microglial cells, which can further amplify ROS production. In addition, levels of antioxidant molecules are relatively low in mature myelinating oligodendrocytes (Giacci et al., 2018). Thus, both mechanisms may further contribute to the vulnerability of these cells to oxidative stress in inflammatory demyelinating diseases.

The case of the *dmy* rat, an autosomal recessive mutant, is very illustrative (Kuwamura et al., 2004). Dmy rats bear a mutation in the mitochondrial magnesium transporter gene (*MRS2*), resulting in the insertion of an 83-bp genomic DNA segment into the *Mrs2* transcript and complete functional inactivation of the mutant allele (Kuramoto et al., 2011). During postnatal myelination, these animals exhibit severe myelin breakdown in combination with abnormal iron accumulation and significant upregulation of iron regulatory proteins in the white matter (Izawa et al., 2010). These changes were detectable early in demyelinating lesions and get worse in parallel with the progression of the disease, suggesting a crucial role of iron accumulation in the phenotype of these mutants (Izawa et al., 2010). Iron accumulation and ferritin upregulation were observed mainly in astrocytes, and a significant upregulation of the antioxidant enzyme heme oxygenase-1 (HO-1, *HMOX1*) was found predominantly in myelinating oligodendrocytes. In contrast to its antioxidant activity, HO-1 may transduce oxidative stress into pathological sequestration of iron in the mitochondria of oligodendrocytes and astrocytes. High levels of iron in the mitochondria of oligodendrocytes and astrocytes may promote further oxidative stress, bioenergetic failure, and thereby perpetuate white matter injury long after the initiating insult has dissipated (Schipper et al., 1991; Schipper, 1999). Thus, in order to understand the pathophysiology of inflammatory demyelinating diseases, it will be essential to study the participation of astrocytes and microglial cells in this process. Exploring how these cells metabolize iron during and after demyelination will be key to design effective therapies and to prevent iron-induced myelin damage in MS.

## The Transferrin Receptor as a Target for Brain Drug Delivery

The pharmacological treatment of neurological diseases is hampered by the low permeability of the BBB to many drugs and molecules. To overcome this obstacle, a promising strategy is to target CNS-active drugs to BBB-associated carriers and receptors, among which the TfR, highly expressed by BCECs (Jefferies et al., 1984), has been extensively studied. TfR-binding molecules can be delivered into the brain in a *Trojan horse*-like fashion (Boado and Pardridge, 2011). Although this approach has allowed accumulation of different drugs in the brain parenchyma, the precise mechanism(s) that mediate drug or nanocarrier release from the TfR and transport into the brain parenchyma remain to be elucidated (Johnsen et al., 2017). The choice of the ligand widely varies across studies, including Tf, antibodies, and peptides which bind to different epitopes of the TfR. Monoclonal antibodies are widely used. Without interfering with the normal binding site of Tf, these antibodies recognize the TfR and bind to it with high affinity (Jefferies et al., 1984, 1985). Like endogenous Tf, OX26 has been tested as a targeting molecule both, as a fusion construct with different drugs, and together with different kinds of nanocarriers (Y. Zhang and Pardridge, 2001; Pardridge, 2005; Y. Zhang and Pardridge, 2006 ; Xia et al., 2007).In addition, some groups have recently developed small peptides that efficiently bind to the TfR (Z. Wang et al., 2015; Kuang et al., 2016). The advantage of these small peptides is that they do not compete with endogenous Tf for their receptor and, more importantly, lack the potential adverse effects generated by the presence of an Fc antibody domain (Couch et al., 2010). However, compared with monoclonal antibodies, these peptides have significantly less affinity for the TfR, and they are unstable in the plasma.

Most of the preclinical data on the use of TfR-targeting as a means for drug delivery into the CNS suggest the concept to be applicable. However, the absolute amount of drug transported via this route remains small, which needs to be solved to achieve clinical use. Thus, different strategies have been proposed to improve current systems and increase drug accumulation in the brain parenchyma, particularly for antibody-based treatments. Modifications to increase drug uptake in the brain require, first, reducing the affinity of the antibody to avoid degradation in the lysosomes and maintain the expression of TfR in the BBB. Second, modifying the avidity of the antibody as the valency of the antibody molecule affects the subsequent expression and intracellular movement of the TfR (Crépin et al., 2010). Therefore, the use of antibodies with a single Fab fragment prevents their degradation in lysosomes without affecting the surface expression of the TfR. Antibodies with two Fab fragments are characterized by greater internalization into BCECs but greater targeting toward lysosomal degradation rather than to transcytosis (Niewoehner et al., 2014). Finally, as iron release in the endosomal compartment requires local acidification (Skjørringe et al., 2015), a pH-sensitive drug delivery system may prove relevant, specifically for Tf-conjugated drugs. This does not necessarily imply, however, that ligand-like antibodies might detach easily, as lowering the pH can significantly modify the receptor structure, limiting ligand binding (Steere et al., 2012). Overall, TfR-mediated brain drug delivery appears as a relevant new intervention to treat CNS diseases more efficiently.

## Final Remarks

Although recent studies have greatly improved our knowledge of brain iron homeostasis, much more research is needed to fully elucidate the participation of oligodendrocytes and astrocytes in brain iron equilibrium. One key challenge for the field will be to define the molecular mechanism of iron incorporation and management in OPCs and mature myelinating oligodendrocytes as well as to identify the primary source of iron for these cells. It will be equally important to understand the mechanisms of iron uptake and efflux in astrocytes during postnatal brain development or under pathological situations such as demyelination. In the near future, the use of new genetical tools for the specific manipulation of iron related proteins specifically in oligodendrocytes or astrocytes will allow us to test in detail the model proposed in Figure 2A. This must be done in the developing brain, during remyelination and finally, in the aging CNS. These data will be useful to intervene and prevent the neurological sequels of iron deficiency during early brain development, to promote remyelination in demyelinating diseases, and to stop demyelination, neurodegeneration, and oxidative stress in the aging brain.
